# Fibroblast Growth Factor Receptor 2c Signaling Is Required for Intestinal Cell Differentiation in Zebrafish

**DOI:** 10.1371/journal.pone.0058310

**Published:** 2013-03-06

**Authors:** Da-Wei Liu, Su-Mei Tsai, Bih-Fen Lin, Yun-Jin Jiang, Wen-Pin Wang

**Affiliations:** 1 Institute of Medical Sciences, Tzu-Chi University, Hualien, Taiwan; 2 Department of Laboratory Medicine and Biotechnology, Tzu-Chi University, Hualien, Taiwan; 3 Division of Molecular and Genomic Medicine, National Health Research Institutes, Zhunan Town, Miaoli County, Taiwan; 4 Department of Molecular Biology and Human Genetics, Tzu-Chi University, Hualien, Taiwan; National Cancer Institute, United States of America

## Abstract

**Background:**

There are four cell lineages derived from intestinal stem cells that are located at the crypt and villus in the mammalian intestine the non-secretory absorptive enterocytes, and the secretory cells, which include mucous-secreting goblet cells, regulatory peptide-secreting enteroendocrine cells and antimicrobial peptide-secreting Paneth cells. Although fibroblast growth factor (Fgf) signaling is important for cell proliferation and differentiation in various tissues, its role in intestinal differentiation is less well understood.

**Methodology/Principal Findings:**

We used a loss of function approach to investigate the importance of Fgf signaling in intestinal cell differentiation in zebrafish; abnormal differentiation of goblet cells was observed when Fgf signaling was inhibited using SU5402 or in the Tg(hsp70l*dnfgfr1-EGFP)* transgenic line. We identified Fgfr2c as an important receptor for cell differentiation. The number of goblet cells and enteroendocrine cells was reduced in *fgfr2c* morphants. In addition to secretory cells, enterocyte differentiation was also disrupted in *fgfr2c* morphants. Furthermore, proliferating cells were increased in the morphants. Interestingly, the loss of *fgfr2c* expression repressed secretory cell differentiation and increased cell proliferation in the *mib^ta52b^* mutant that had defective Notch signaling.

**Conclusions/Significance:**

In conclusion, we found that Fgfr2c signaling derived from mesenchymal cells is important for regulating the differentiation of zebrafish intestine epithelial cells by promoting cell cycle exit. The results of Fgfr2c knockdown in *mib^ta52b^* mutants indicated that Fgfr2c signaling is required for intestinal cell differentiation. These findings provide new evidences that Fgf signaling is required for the differentiation of intestinal cells in the zebrafish developing gut.

## Introduction

In adult mammals, the epithelium of the small intestine comprises two structures: finger-like villi and pocket-like crypts of Lieberkühn. Intestinal stem cells are located at the bottom of the crypt. Crypts also contain transit amplifying progenitor cells. These proliferating cells differentiate, then migrate to villi and are removed at the top of the villi by apoptosis. There are four cell lineages that derive from intestinal stem cells: the non-secretory absorptive enterocytes, and secretory cells, which include mucous-secreting goblet cells, regulatory peptide-secreting enteroendocrine cells, and antimicrobial peptide-secreting Paneth cells [1], [2], [3], [4]. It has been reported that, unlike mammals, zebrafish do not possess crypts of Lieberkühn or Paneth cells [5].

Many signaling molecules regulate stem cell self-renewal, proliferation, and differentiation in the intestines [6], [7]. The Wnt pathway is important in controlling crypt cell proliferation. The crypt precursors of *Tcf4* null mice exhibit decreased cell proliferation, and comprise various differentiated cells [8]. However, in mice that lack *Apc* expression (*APC^min^*), crypt cells exhibit greater proliferation than they do in *Tcf4* null mice, and in the deficient mice, these cells only differentiate to form Paneth cells [9], [10]. In *Apc* mutant zebrafish (*Apc^mcr^*), the enterocyte differentiation marker, *intestinal fatty acid binding protein* (*ifabp*), was failed to express [11], [12]. In *bone morphogenetic protein receptor 1a* (*Bmpr1a*) mutant mice or *Noggin* transgenic mice, the expansion of proliferating cells in the crypt results in intestinal polyposis [13], [14]. Three secretory cells are also reduced in Bmpr1a mutant mice [15]. Interestingly, Wnt signaling is highly activated in these Bmp pathway deficient mice. Additionally, Notch signaling is important for cell lineage commitment and proliferation. *Notch1* and *Notch2* double knockout mice exhibit complete conversion of proliferating crypt progenitors into post-mitotic goblet cells [16]. In *deltaD* (*aei^AR33^*) and *mind bomb* (*mib^ta52b^*) mutation zebrafish, secretory cells are also overproduced [17]. Interestingly, concerted activation of Notch and Wnt pathways are required for the maintenance of undifferentiated and proliferative cells in crypts. *Hes1* is highly expressed in undifferentiated cells of *Apc^min^* mice. Notch signaling inhibitor can induce reduction in the number of proliferated cells and increase differentiation into goblet cells in *Apc^min^* mice [18].

Fibroblast growth factor (Fgf) signaling is involved in intestinal development and cell differentiation. There are 22* Fgfs* and 4 *Fgfrs* in mice [19], [20]. Fgfr1∼3 has two isoforms, b and c, which result from alternative splicing. These two isoforms have different ligand-binding specificities [21]. Fgf10 signaling is required, in a dose-dependent manner for the survival and proliferation of colonic epithelia progenitor cells [22]. Overexpression of Fgf10 can attenuate stomach and duodenum cell differentiation [23], [24]. Goblet cells, but not Paneth cells or enteroendocrine cells, were increased in recombinant FGF7 protein treated rats [25]. Furthermore, the depth of the crypt and the numbers of proliferating cells were increased in *Fgfr3* deficient mice but villi length and the distribution of differentiated intestinal cells were unaffected [26]. Nevertheless, a recent report indicated that Paneth cell differentiation is reduced in *Fgfr3* deficient mice [27]. These evidences suggest that the Fgf signaling pathway has a regulatory role in cell differentiation in the gastrointestinal tract. However, few reports address how Fgf signaling controls intestinal cell differentiation.

Zebrafish offer many advantages for studying intestinal cell differentiation, they have rapid development and transparent embryos, and techniques for the manipulation their gene expression are well-established. Furthermore, the early-stage development of the zebrafish gastrointestinal tract has been well described [5], [28], [29]. Using *Tg(hsp70l:dnfgfr1-EGFP)* transgenic fish to inhibit Fgf signaling [30], we found that heat-shock treatments inhibited the differentiation of secretory cells. We further found that Fgfr2c signaling controls the differentiation of secretory and absorptive cells by regulation of progenitor cell proliferation. These findings provide new evidence that Fgf signaling is required for the differentiation of intestinal cells in the zebrafish developing gut.

## Materials and Methods

### Ethics Statement

All embryos were handled according to protocols approved by the Institutional Animal Care and Use Committee of Tzu Chi University, Hualien, Taiwan (approval ID: 99056).

### Zebrafish

Zebrafish (*Danio renio*) were raised as described in the Zebrafish Book [31]. The AB wild type (WT) strain was used for morpholino injection and other experiments. The transgenic and mutant fish lines used in this study were *Tg(hsp70l:dnfgfr1-EGFP)^pd1^*, provided by Taiwan Zebrafish Core Facility at Academia Sinica, TZCAS, and *mib^ta52b^*, respectively.

### SU5402 Treatment and Heat Induction Experiments

SU5402 (Calbiochem, UK) was dissolved into DMSO. Three days post fertilization (dpf) embryos were placed in a 6-well plate and SU5402 solution (2.5 or 3.4 µM) was added. We used 0.025% DMSO solution as control. At 4 dpf, the solution was replaced with fresh solution of the same concentration. All treated embryos were harvested at 5 dpf.

Water was pre-warmed to 37°C. Zebrafish embryos received a single 37°C heat-shock treatment for one hour in an air incubator, and the water then left to cool to 28°C.

### Microinjection

All morpholinos (MOs) were obtained from Gene Tools, LLC. MOs targeting *fgfr2b*, *fgfr2c*, *fgfr4*, and with a 5-base mismatch for *fgfr2c* MO were used in this study. MOs sequences were as follows: *fgfr2b* MO 5′-CGCTCCTGCTTTTTTACCTGGTATG-3′, *fgfr2c* MO 5′-AAGCAGTGGAAGGTGAGTTTATACC-3′, 5-base mismatch for *fgfr2c* MO 5′-AAcCAcTGcAAGGTcAcTTTATACC-3′ [32]; *fgfr4-I1E2* MO 5′-ATATCTGCTGGAGTAAAAAATGAGG-3′ [33]. MOs (4 ng) were injected into 1–2 cell stage embryos.

### RT-PCR and qPCR Analyses

The whole gut was collected at 5 dpf using #5 forceps for RNA extractions. Total RNA was extracted using TRIZOL Reagent (Invitrogen, Carlsbad, CA, USA). DNase-I treated total RNA (5 µg) was used to generate cDNA using an ImProm-II kit (Promega, Madison, WI, USA). PCR primers used in this study are listed in Table S1.

For qPCR, reverse transcription reaction was performed using a GoScript kit (Promega, Madison, WI, USA). Expression level of *pepT1* was detected according to the published primer sequences [34]. Expression of *β-actin1* was detected as an internal control [35]. The primer sequence for *pepT1* was F-AACACAAACATCAAGCAAACC and R-AACTACCAACCCTCAAGCCC. The primer sequence for *β-actin1* was F-GCTGTTTTCCCCTCCATTGTT and R-TCCCATGCCAACCATCACT. The Maxima SYBR Green qPCR Master Mix (Fermentas International Inc., Ontario, Canada) system was used to quantify the transcripts.

### Whole Mount in situ hybridization

The following in situ probes were used: *agr2*, *glucagon*, and *pepT1* [34]; *fgfr1–4* [36], and *ifabp*. The *ifabp* fragment was amplified by PCR and subcloned into pGEM-T vector (Promega). The DIG-labeled probes were generated by in vitro transcription using a DIG RNA labeling kit (Roche). For whole mount in situ hybridization, DIG-labeled probes were used to hybridize the embryos overnight at 65°C, and were then washed under high stringency condition. Embryos were treated with blocking buffer (Roche) and incubated with AP-conjugated anti-DIG antibody overnight at 4°C (1∶2000, Roche). Embryos were washed to remove excess antibody and then colored with NBT/BCIP (Roche).

### Whole Mount Wheat Germ Agglutinin (WGA), Immunofluorescence Staining and BrdU Labeling

For WGA staining, embryos were fixed in 4% paraformaldehyde at 4°C overnight, and the yolks were removed before staining. Fixed embryos were washed three times for 5 min with PBST (containing 0.3% TritonX-100) and buffer B (10 mM HEPES and 0.15 M NaCl, pH = 7.5), and then incubated with rhodamine conjugated WGA (Vector Laboratories, 1∶100, in buffer B) at 4°C overnight. After washing with PBST (four times for 30 min), embryos were mounted in SlowFade Gold antifade reagent with DAPI (Molecular Probes, S-36938). We counted the WGA positive cells for all the embryos using ImageJ software.

For WGA and 2F11 double labeling, embryos were blocked in PBST, containing 4% BSA, for one hour after WGA staining. Embryos were then incubated in mouse monoclonal 2F11 antibody (1∶1000, a gift from Dr. Julian Lewis) at 4°C overnight. After washing with PBST as described above, embryos were incubated in goat anti-mouse IgG Alexa Fluor 488 (Molecular Probes, A-11029, 1∶100) at 4°C overnight. After washing with PBST, embryos were rinsed with PBS three times, and the embryos were nuclear counterstained with TOPRO-3 (Molecular Probes, T-3605, 1∶1000). Embryos were mounted in SlowFade Gold antifade reagent with DAPI.

For histone H3-phosphorylated (H3P) staining, we used H3P antibody (Santa Cruz, sc-8656-R, 1∶200) and goat anti-rabbit IgG Hilyte Fluor 488 (AnaSpec, 1∶100) or goat anti-rabbit IgG Alexa Fluor 546 (Molecular Probes, A-11035, 1∶200). Before blocking, embryos were rinsed twice with sodium citrate buffer (10 mM, pH = 6). Embryos then were incubated in citrate buffer at 95°C for 5 min.

For BrdU labeling, 3 or 5 dpf embryos were incubated in 10 mM BrdU solution at 6°C for 30 min. Embryos were then rinsed five times and incubated with Ringer’s solution (0.116 M NaCl, 1.8 mM CaCl_2_, 2.9 mM KCl, and 5 mM HEPES) at 28°C for 1 h. Antigen retrieval was performed using 2 N HCl at 37°C for 20 min. Monoclonal anti-BrdU antibody (Sigma, B2531; 1∶200) was used to detect labeled cells. Goat anti-mouse IgG Alexa Fluor 488 antibody was used as described above.

For counting cell number, longitudinal confocal images, including esophagus to anus, were collected using a LEICA TCS SP2 AOBS confocal microscope. Stacked images were analyzed with ImageJ software Plugin (Cell Counter). The WGA, 2F11, BrdU, and H3P positive cells in intestinal epithelial layer were counted manually using this software. The two-tailed Student’s *t*-test was used for analyzing changes in cell number.

### Whole Mount TUNEL Assay

Fixed embryos with their yolk removed were incubated with proteinase K (100 µg/ml) at 28°C for 35 min. After rinsing with PBS, embryos were incubated with TUNEL reaction mixture from an In Situ Cell Death Detection Kit, TMR red (Roche) at 37°C for 1 h. Labeled embryos were washed with PBST four times for 15 min. Nuclear counterstaining and mounting was performed as described above.

## Results

### Secretory Cells were Reduced after Inhibition of Fgf Signaling

**Figure 1:**
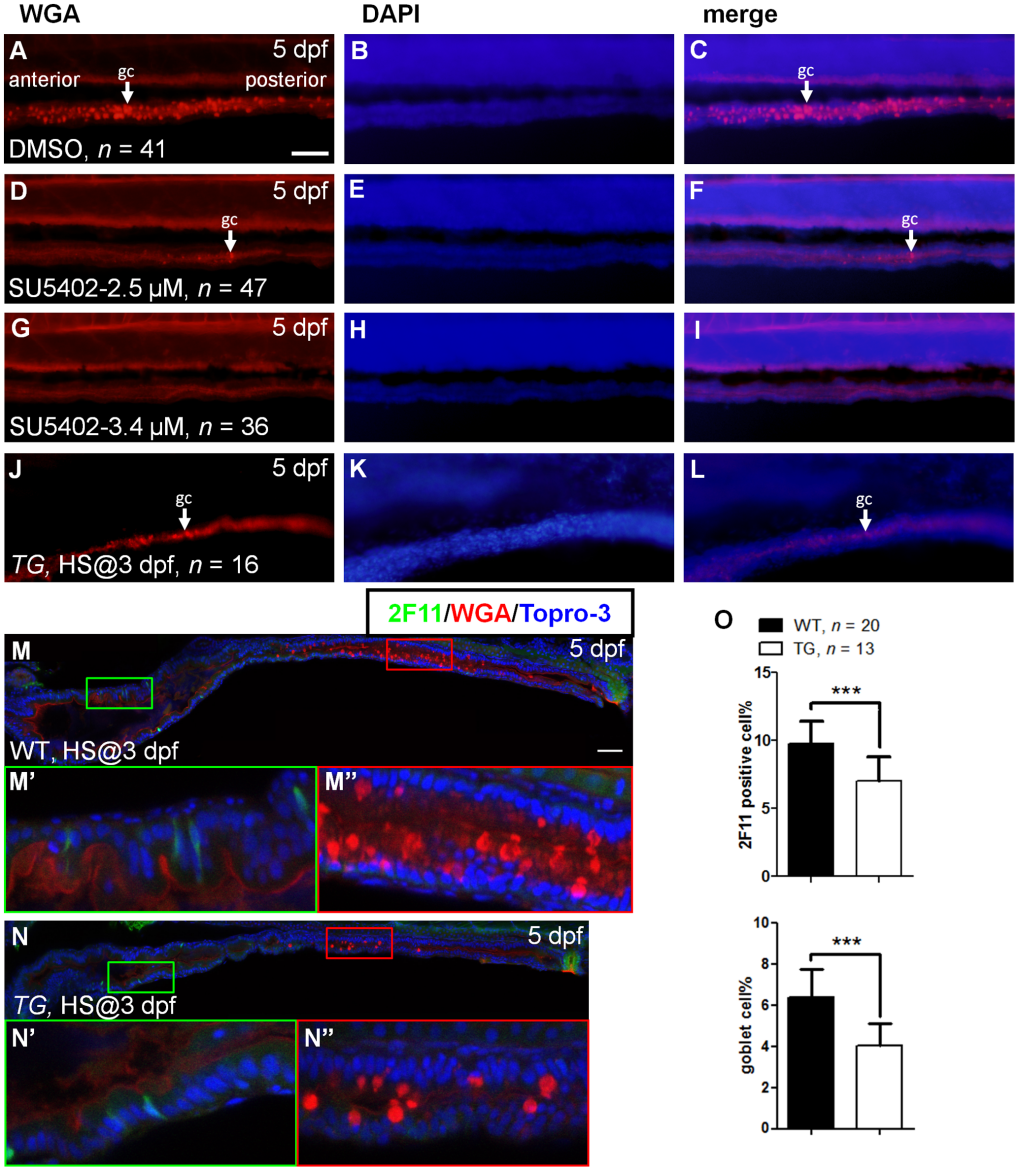
Effects on cell differentiation after inhibition of Fgf signaling. Five dpf **e**mbryos were stained with WGA after incubation in (**A–C**) DMSO, (**D–F**) SU5402 (2.5 µM), and (**G–I**) SU5402 (3.4 µM) at 3 dpf. (**J–L**) Three dpf *Tg(hsp70l:dnfgfr1-EGFP)* embryos were heat treated, then stained with WGA at 5 dpf. White arrow indicated the goblet cell (gc). DAPI nuclear counter stain showed the tissue structure. (**M**) Heat-treated WT embryos were used as controls. (**N**) Heat shocked transgenic embryos were double labeled with WGA and 2F11 antibody at 5 dpf. (**M’,N’**) The magnified image shows enteroendocrine and (**M’’,N’’**) goblet cells. (**O**) The bar charts show the percentage of 2F11 or WGA positive cells. All images were lateral view with anterior at left and posterior at right. Error bars indicate SD. Scale bars = 50 µm.

To determine the role of Fgf signaling in the differentiation of zebrafish intestinal cells, we treated wild type embryos with SU5402 and used the *Tg(hsp70l:dnfgfr1-EGFP)* fish line to inhibit Fgf signaling activity, respectively. In zebrafish embryos, intestinal tract compartmentalizes into three segments: intestinal bulb, mid-intestine, and posterior intestine. The mucin-secreting goblet cells are presented in the mid-intestine at 5 dpf [5]. We first analyzed the differentiation of goblet cells by using rhodamine conjugated WGA to stain the cells’ mucin [28]. In 5 dpf control embryos, goblet cells could be detected at the mid-intestine (Fig. 1A–1C, 93.0±3.4 goblet cells/embryo). When embryos were treated with SU5402 (2.5 µM) from 3 dpf, goblet cells were greatly reduced in number (Fig. 1D–1F, 3.3±0.9 goblet cells/embryo) at 5 dpf. There was a dosage dependent effect after treatment at the greater dose of 3.4 µM SU5402 (Fig. 1G–1I, 0.7±0.2 goblet cells/embryo). In addition to pharmacological inhibition, we also provided a genetic evidence using *Tg(hsp70l:dnfgfr1-EGFP)* fish line, which is widely used to block all Fgf signaling [30], [37], [38], [39], [40], [41], [42]. We have used this fish line to study Fgf signaling in liver homeostasis [43]. The *Tg(hsp70l:dnfgfr1-EGFP)* fish line showed a similar response when embryos were heat treated at 37°C for 1 hour at 3 dpf, and analyzed at 5 dpf. Goblet cells were greatly reduced in heat treated embryos (Fig. 1J–1L).

We examined whether the differentiation of enteroendocrine and goblet cells was affected in Fgf signaling inhibited embryos, by using 2F11 antibody to detect secretory cells [17]. After whole mount immunostain using 2F11 antibody, WGA staining, and Topro-3 nuclear counter staining, embryos were analyzed by confocal microscope. Scattered 2F11 positive cells were present in WT controls, particularly in the intestinal bulb (Fig. 1 M and 1 M’). As expected, goblet cells were present in the mid-intestine (Fig. 1 M and 1 M’’). After heat treatment of embryos from *Tg(hsp70l:dnfgfr1-EGFP)*, scattered 2F11 positive cells were reduced in number (Fig. 1N and 1N’). Goblet cell numbers were greatly reduced (Fig. 1N and 1N’’). We further quantified the proportion of 2F11 and WGA positive cells in total intestinal epithelia. In control embryos, 9.8±1.6% of cells were positive for 2F11. Fewer 2F11 positive cells were observed in Fgf signaling inhibited embryos (7.0±1.8%, *P*<0.001; Fig. 1O). For goblet cells, 4.0±1.1% of WGA positive cells were present in Fgf signaling inactivated embryos, but were fewer than 6.4±1.4% in control embryos (*P*<0.001; Fig. 1O). These observations indicate that Fgf signaling is important for intestinal secretory cell differentiation.

### Fgfr2c Signaling is Required for Secretory Cell Differentiation

**Figure 2:**
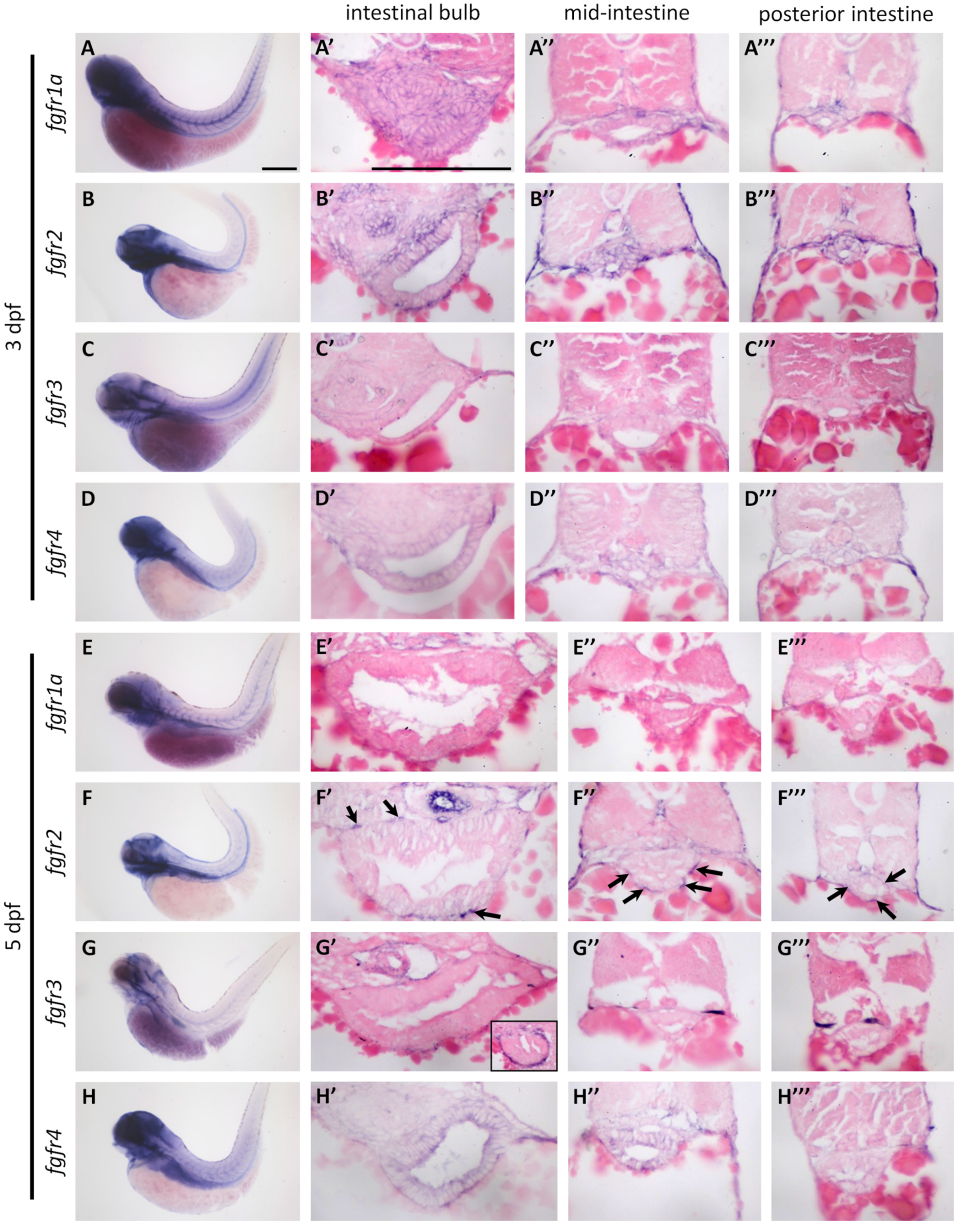
The expression pattern of zebrafish *fgfr* genes. The expression of *fgfr1a-4* was analyzed by WISH: (**A–D**) at 3 dpf and (**E–H**) at 5 dpf. WISH embryos were sectioned and analyzed for expression of *fgfr1a-4* in: (**A’–H’**) the intestinal bulb, (**A’’–H’’**) the mid-intestine, and (**A’’’–H’’’**) the posterior intestine. Black arrows indicated the *fgfr2* expression in mesenchymal cells. Scale bars = 200 µm.

There are five *fgf* receptor genes, *fgfr1a, fgfr1b, fgfr2, fgfr3*, *and fgfr4*, in zebrafish [36], [44]. To identify receptors important for intestinal cell differentiation, we used RT-PCR to analyze *fgfrs* expression in the whole gut, including the esophagus, intestine, liver, and pancreas at 5 dpf. Except for *fgfr1b*, four receptors were detected in gut cDNA (Fig. S1A). We used whole mount in situ hybridization (WISH) to analyze the temporal and spatial expression pattern of the four receptors. Expression of *fgfr1a*, *fgfr2*, and *fgfr4* were detected in the intestine at 3 dpf (Fig. 2A, 2B, and 2D). To analyze the cellular localization of these receptors, specimens were sectioned in paraffin. The *fgfr1a* and *fgfr2* genes were expressed in the mesenchymal and epithelial layers of the intestinal bulb, mid-intestine, and posterior intestine at 3 dpf (Fig. 2A’-2A’’’ and 2B’-2B’’’). The *fgfr4* gene was expressed in the epithelium in low levels at 3 dpf (Fig. 2D’-2D’’’). However, *fgfr3* was not expressed in the intestine at 3 dpf (Fig. 2C–2C’’’) or 5 dpf (Fig. 2G–2G’’’), although many signals were present in mesenchymal cells of the esophagus at 5 dpf (inset in Fig. 2G’). Expression of *fgfr1a* was no longer expressed in intestine but in pancreatic islet at 5 dpf (Fig. 2E–2E’’’ and data not shown). Only *fgfr2* and *fgfr4* were expressed in the intestine at 5 dpf (Fig. 2F and 2H). The *fgfr2* was strongly expressed in intestinal mesenchymal cells, but not in epithelium cells at 5 dpf (Fig. 2F’–2F’’’, arrows). Expression of *fgfr4* was more intensive in the intestinal epithelium at 5 dpf than they were at 3 dpf (Fig. 2H’–2H’’’).

**Figure 3:**
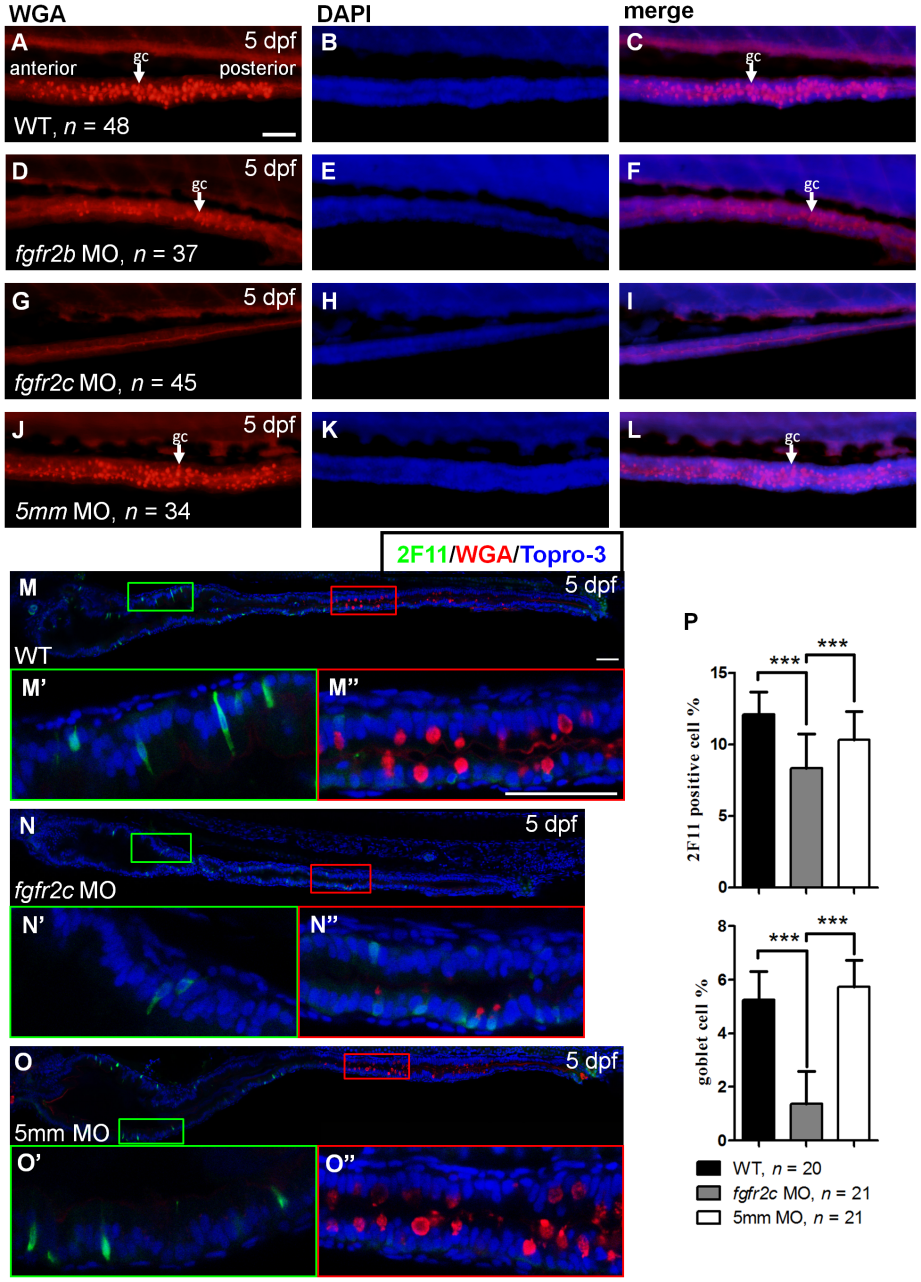
The secretory cell differentiation of *fgfr2c* morphants. WGA staining was performed on 5 dpf (**A–C**) WT embryos, (**D–F**) *fgfr2b*, (**G–I**) *fgfr2c*, and (J–L) *fgfr2c-5* *mm* morphants. White arrows indicated goblet cell (gc). Double labelling using 2F11 antibody and WGA in (**M**) WT embryos, (**N**) *fgfr2c* morphants, and (**O**) *fgfr2c-5* *mm* morphants. The magnified image shows (**M’–O’**) enteroendocrine cells and (**M’’–O’’**) goblet cells. Topro-3 was used for nuclear counter staining (blue). (**P**) The bar charts show the percentages of 2F11 or WGA positive cells. All images were lateral view with anterior at left and posterior at right. Error bars indicate SD. Scale bars = 50 µm.

Because *fgfr2* was the only receptor that was strongly expressed in the intestine at both 3 and 5 dpf, we analyzed whether intestinal cell differentiation was disrupted in Fgfr2 signaling inactive embryos. Two *fgfr2* isoforms, *fgfr2b* and *fgfr2c*, have been identified [36]. We used morpholino antisense oligonucleotides to knockdown gene expression in zebrafish embryos. Two morpholinos, which respectively, target *fgfr2b* and *fgfr2c*, have been used by us to study zebrafish embryo development. One *fgfr2c*-*5* *mm* morpholino, which contained 5 mismatch bases in *fgfr2c* morpholino sequence, was used as a control [32]. We studied intestinal cell differentiation in the morpholino-injected morphants. The number of goblet cells was reduced in *fgfr2b* morphants compared to WT (Fig. 3A–3F). Moreover, goblet cell differentiation was almost completely absent from the *fgfr2c* morphants (Fig. 3G–3I). Goblet cell was normally differentiated in *fgfr2c*-*5* *mm* morphants (Fig. 3J–L).

We quantified the proportion of secretory cell in *fgfr2b* and *fgfr2c* morphants after 2F11 immunostaining and WGA staining. Goblet cell numbers were slightly reduced in the *fgfr2b* morphants, similar to our WGA staining results (Fig. S1B–1D; 5.4±1.45% in WT and 3.7±1.05% in morphants, *P*<0.001). The percentage of 2F11 positive cells was not reduced in *fgfr2b* morphants (Fig. S1B–1D; 10.9±2.33% in WT and 11.4±1.64% in morphants, *P = *0.7995). In comparison with *fgfr2b* morphants, secretory cell differentiation was more significantly inhibited in *fgfr2c* morphants. Goblet cells were greatly reduced in number in *fgfr2c* morphants (Fig. 3M–3P; 1.37±1.21% in *fgfr2c* morphants, 5.72±1.00% in *fgfr2c*-5 mm morphants, and 5.25±1.06% in WT, *P*<0.001). The amount of 2F11 positive cells was reduced in *fgfr2c* morphants compared with control embryos (Fig. 3M–3P; 8.32±2.41% in *fgfr2c* morphants, 10.34±1.97% in *fgfr2c*-5 mm morphants, and 12.08±1.58% in WT, *P*<0.001). Consistent with the results for *Tg(hsp70l:dnfgfr1-EGFP)* embryos, these findings indicated that Fgfr2c signaling is important for secretory cell differentiation.

**Figure 4:**
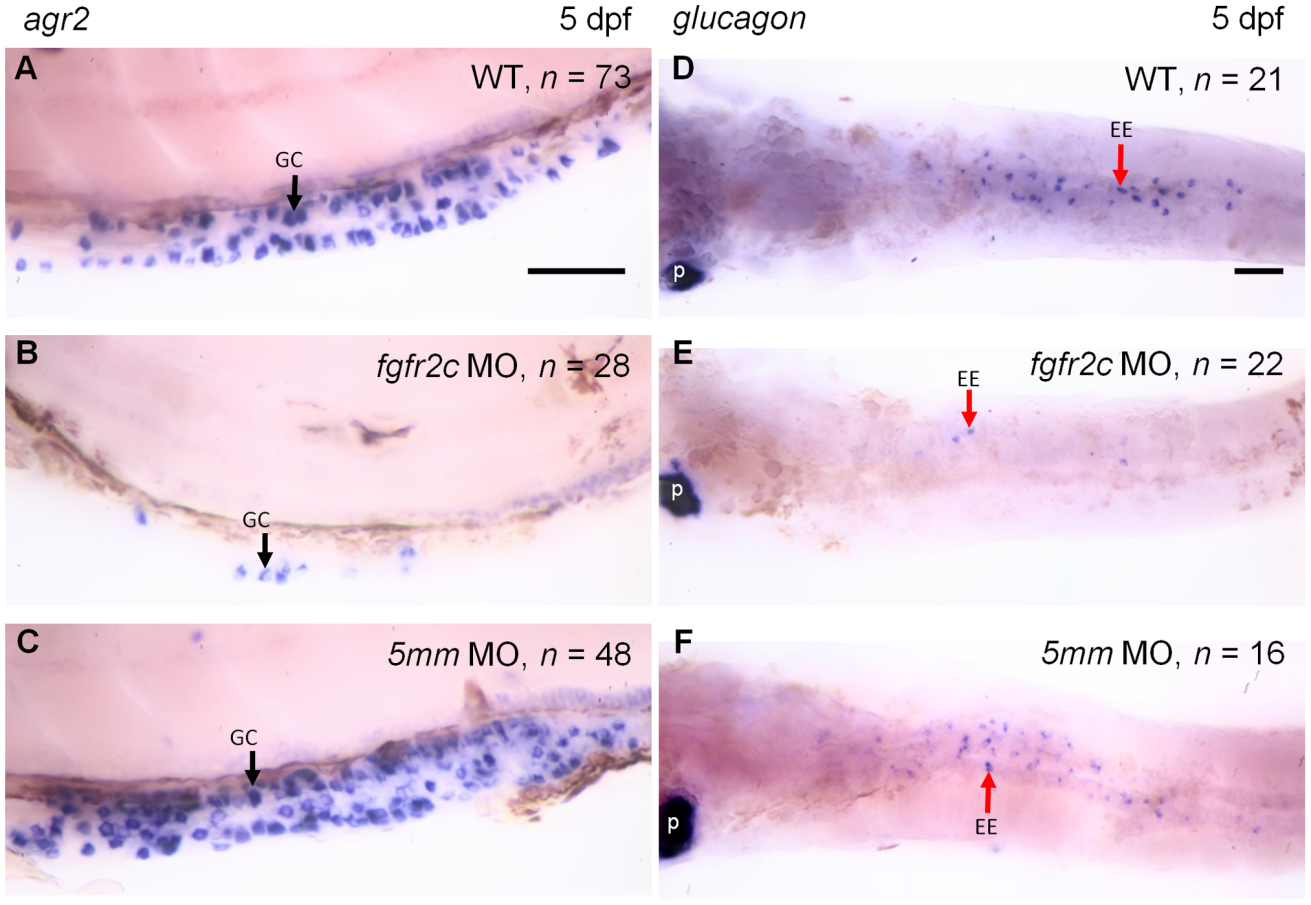
The expression of *agr2* and *glucagon* in *fgfr2c* morphants. WISH of *agr2* (**A–C**, lateral view) and *glucagon* (**D–F**, ventral view) were used to analyze goblet cell and enteroendocrine differentiation, respectively, in 5 dpf embryos. Black arrows indicated the goblet cells (gc) and red arrows indicated the enteroendocrine cells (ee). p: Pancreatic alpha cells. Scale bars = 50 µm.

The loss of WGA signal might also be resulted from downregulation of mucin production. We used a goblet cell specific marker gene, *anterior gradient 2* (*agr2*) to analyze goblet cell differentiation [45], [46]. The WISH results showed that *agr2* expressing cells were presented in mid-intestine of control embryos (Fig. 4A and 4C). In *fgfr2c* morphants, *agr2* expressing cells were dramatically reduced in number (Fig. 4B). These results indicated that goblet cell differentiation was inhibited in *fgfr2c* morphants.

We further used *glucagon* probe to analyze the differentiation of enteroendocrine [5], [34], [47]. The signal could be detected in pancreatic alpha cells in *fgfr2c* morphants and control embryos (Fig. 4D–4F). In contrast, glucagon-expressing cells were greatly reduced in intestine of *fgfr2c* morphants compared with control embryos (Fig. 4D–4F, arrow). Taken together, the differentiation of both secretory cell types was disrupted in *fgfr2c* morphants.

### Enterocyte Differentiation was Disrupted in *fgfr2c* Morphants

**Figure 5:**
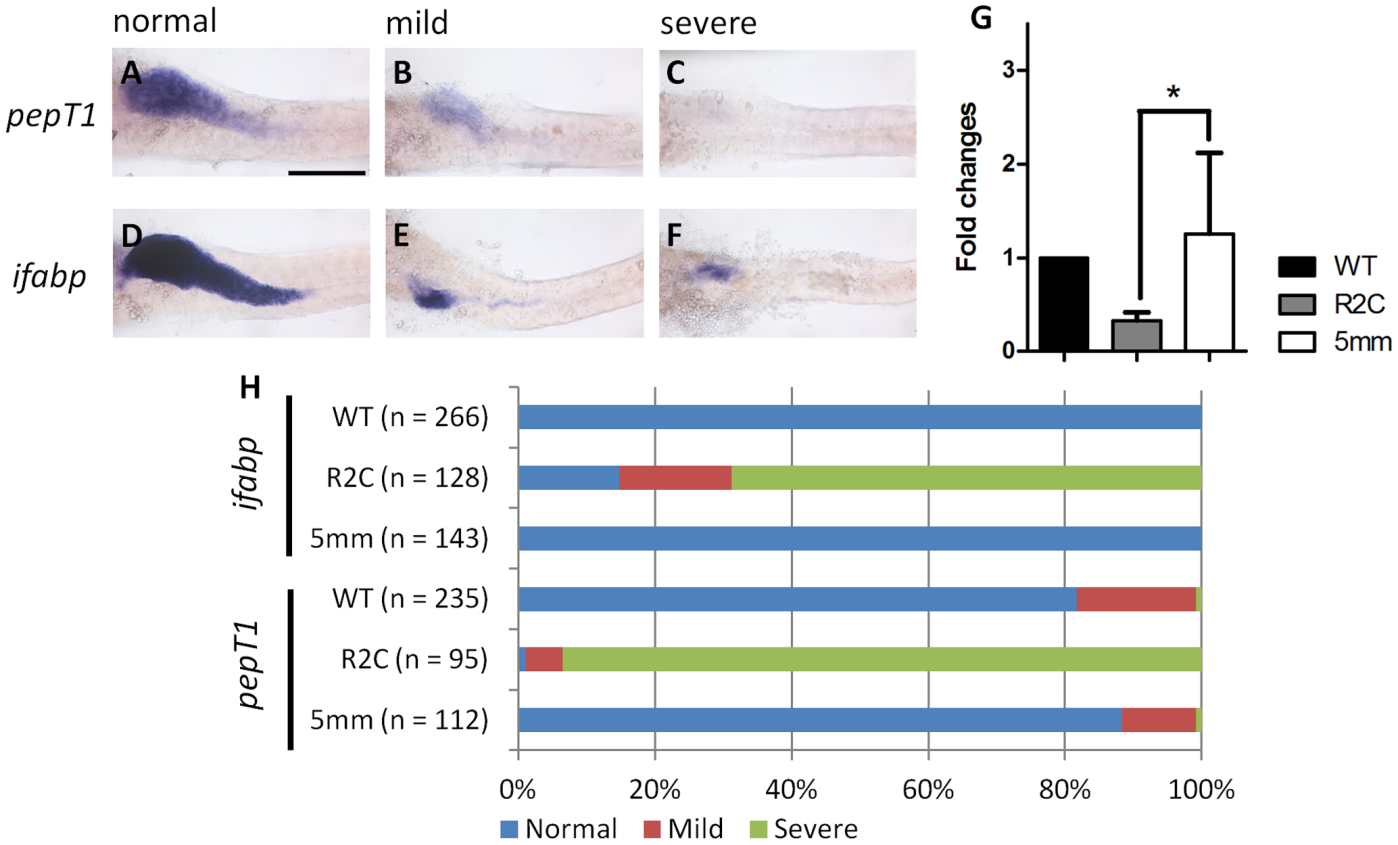
Absorptive cell differentiation in *fgfr2c* morphants. The *pepT1* and *ifabp* WISH were used to analyze enterocyte differentiation in 5 dpf embryos. Three different signal levels were classified as (**A,D**) normal, (**B,E**) mild, and (**C,F**) severe. (**G**) qPCR analysis of the *pepT1* expression level in the *fgfr2c* morphants and control embryos. Data were normalized with *β-actin1* and expressed as fold-induction relative to wild type embryos. Error bars indicate SD. *indicates *P*<0.05. (**H**) The bar charts show the percentages of three expression levels of these two genes in WT embryos, and *fgfr2c* and *fgfr2c-5* *mm* morphants. Scale bar = 200 µm.

We wondered whether the differentiation of absorptive cells was normal in *fgfr2c* morphants. We analyzed the expression of the gene for *intestinal fatty acid binding protein ifabp*, and *oligopeptide transporter pepT1*. These two genes were expressed in the differentiated enterocytes [34]. According to the expression pattern in 5 dpf embryos, three different levels of *pepT1* and *ifabp* gene expression were classified (Fig. 5A–5F): level 1; expression in the whole intestinal bulb (normal; Fig. 5A and 5D). Level 2; expression in the whole intestinal bulb, but at reduced levels (mild; Fig. 5B and 5E). Level 3; expression only in the anterior intestinal bulb or no expression (severe; Fig. 5C and 5F). The *ifabp* expression level was normal in all WT embryos (Fig. 5H). For *pepT1* expression, 81.7% WT embryos were normal. However, 17.4% expressed mild and 0.9% expressed severe patterns (Fig. 5H). The expression of *pepT1* and *ifabp* in *fgfr2c*-5 mm morphants was similar to that of WT embryos (Fig. 5H). However, severe reduction of the enterocyte population was found in most *fgfr2c* morphants. We observed 14.8% morphants with normal, 16.4% morphants with mild, and 68.8% morphants exhibiting severe *ifabp* expression pattern. Reduction in *pepT1* expression was even more obvious. Only 1.1% morphants exhibited the normal *pepT1* pattern. Mild and severe patterns were observed in 5.3% and 91.6% morphants, respectively (Fig. 5H). The results of qPCR also showed the expression level of *pepT1* was reduced in the *fgfr2c* morphants compared with control embryos (Fig. 5G). Therefore, we conclude that the differentiation of absorptive enterocytes was also affected in *fgfr2c* morphants.

### Proliferating Cells were Increased in *fgfr2c* Morphants

**Figure 6:**
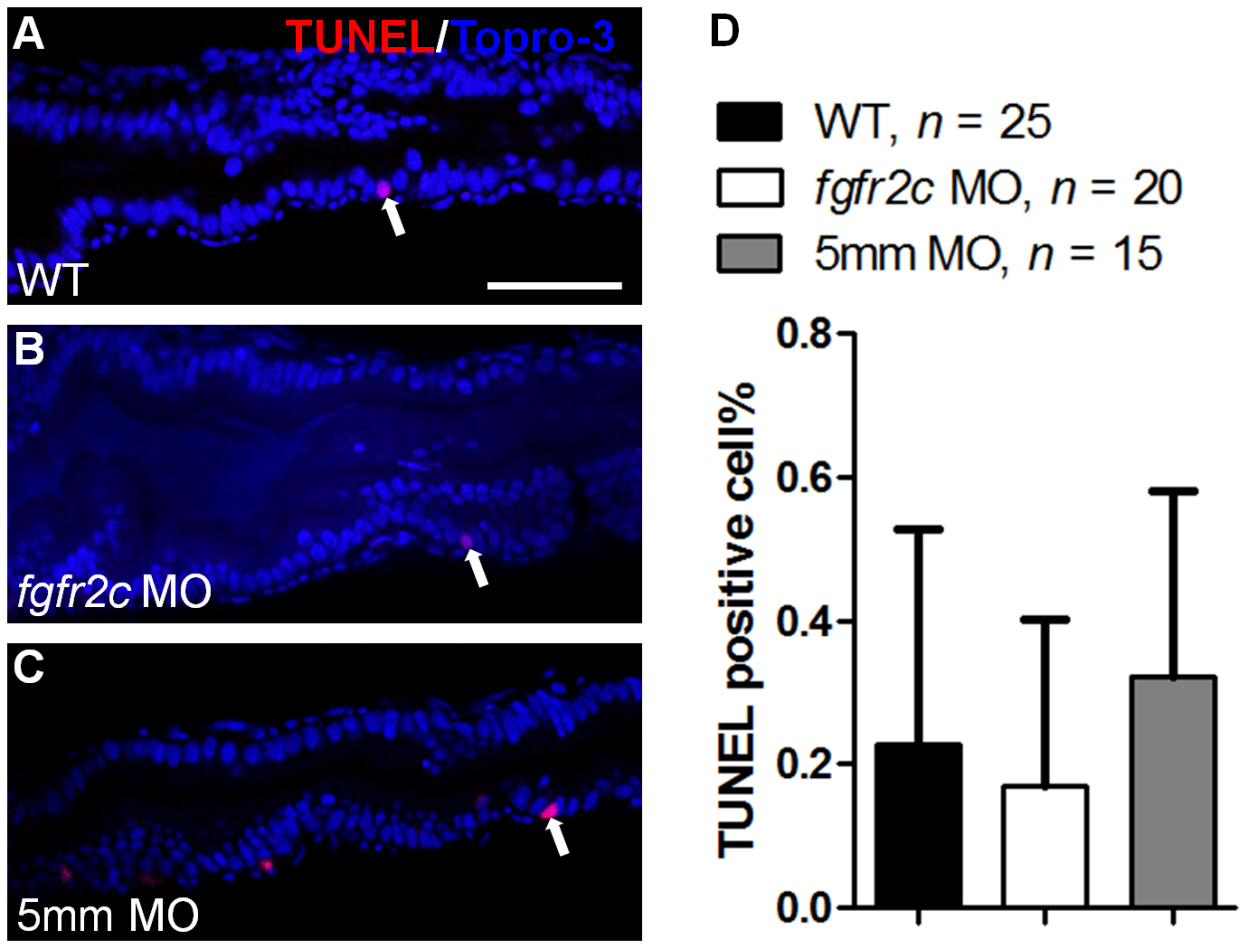
Cell death in *fgfr2c* morphants. TUNEL assay was performed on (**A**) WT embryos, (**B**) *fgfr2c* morphants, and (**C**) *fgfr2c-5* *mm* morphants at 5 dpf. TUNEL-positive cells (red) were detected in developing gut (indicated by white arrows). Topro-3 was used for nuclear counter staining (blue). (**D**) The bar charts show the percentages of TUNEL-positive cells in WT embryos, and *fgfr2c* and *fgfr2c-5* *mm* morphants. Error bars indicate SD. Scale bar = 50 µm.

Because differentiation of both secretory and absorptive cells in the intestine was disrupted, we wondered whether cell death was altered in *fgfr2c* morphants. TUNEL assay indicated that only very few cell deaths occurred in 5 dpf WT embryos, and *fgfr2c* and *fgfr2c*-5 mm morphants (0.2±0.3%, 0.2±0.2%, and 0.3±0.3%, respectively; Fig. 6). Thus, the observed reduction in cell differentiation was not caused by cell death.

**Figure 7:**
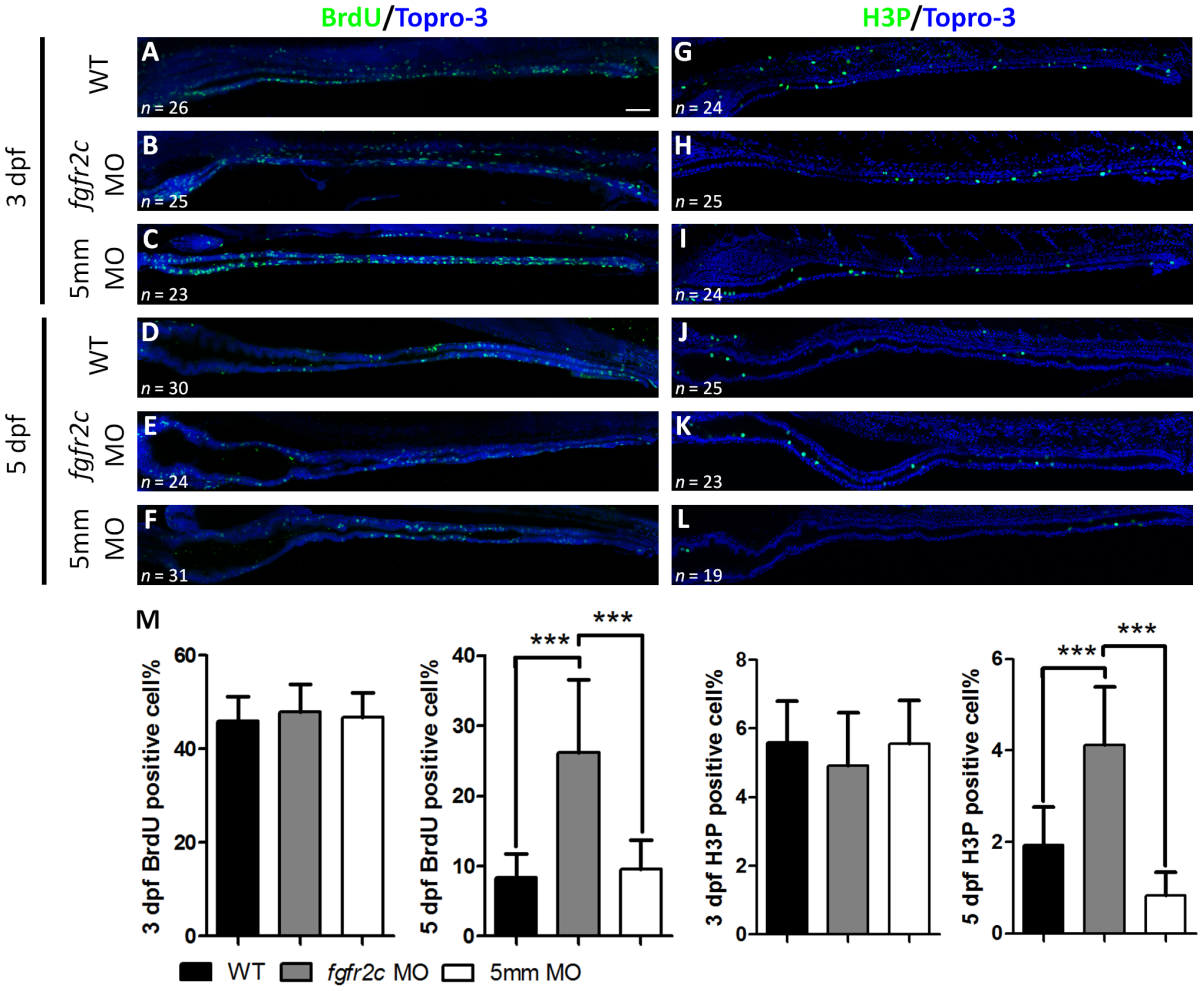
Cell proliferation in *fgfr2c* morphants. The S-phase proliferating cells of WT, *fgfr2c*, and *fgfr2c-5* *mm* morphants were labeled with BrdU at (**A–C**) 3 dpf and (**D–F**) 5 dpf. H3P antibody was used to label M-phase cells at (**G–I**) 3 dpf and (**J–L**) 5 dpf. (**M**) Topro-3 was used for nuclear counter staining (blue). The bar charts show the percentages of proliferating cells. Error bars indicate SD. Scale bar = 50 µm.

We analyzed the cell proliferation status in *fgfr2c* morphants. BrdU incorporation was used to detect the proportion of S-phase cells, and H3P antibody was used to detect M-phase cells, in 3 and 5 dpf embryos. Normally proliferating cells are densely scattered throughout the intestine at 52–74 hours post fertilization (hpf) and reduced in number at 74–120 hpf [5]. We observed 45.9±5.3% S-phase, and 5.6±1.6% M-phase cells in the intestine of WT embryos at 3 dpf (Fig. 7A and 7G). The proportion of cycling cells present in *fgfr2c*-5 mm morphants at 3 dpf was similar to that for WT (S phase: 46.9±5.1%, and M phase: 5.6±1.3%; Fig. 7C and 7I). The percentages of proliferating cells in *fgfr2c* morphants were similar to those of the control embryos at 3 dpf (S phase: 47.6±5.8% and M phase: 4.9±1.6%; Fig. 7B, 7H, and 7 M). The cycling cells of WT embryos were significantly reduced in number at 5 dpf (S phase: 8.4±3.4% and M phase: 1.9±0.8%; Fig. 7D and 7J). A similar reduction occurred in *fgfr2c*-5 mm morphants at 5 dpf (S phase: 9.6±4.2% and M phase: 0.8±0.5%; Fig. 7F and 7L). Although cycling cells were reduced at 5 dpf compared to 3 dpf in *fgfr2c* morphants, the proportion of proliferating cells was still much greater than that of control embryos (S phase: 26.2±10.3% and M phase: 4.1±1.3%, *P*<0.001; Fig. 7E, 7K, and 7M). In summary, cycling cells were unchanged at 3 dpf but increased in number at 5 dpf after inhibition of Fgfr2c signaling.

### Disruption of Cell Differentiation and Proliferation in *mib^ta52b^* Mutants with *fgfr2c* Knockdown

**Figure 8:**
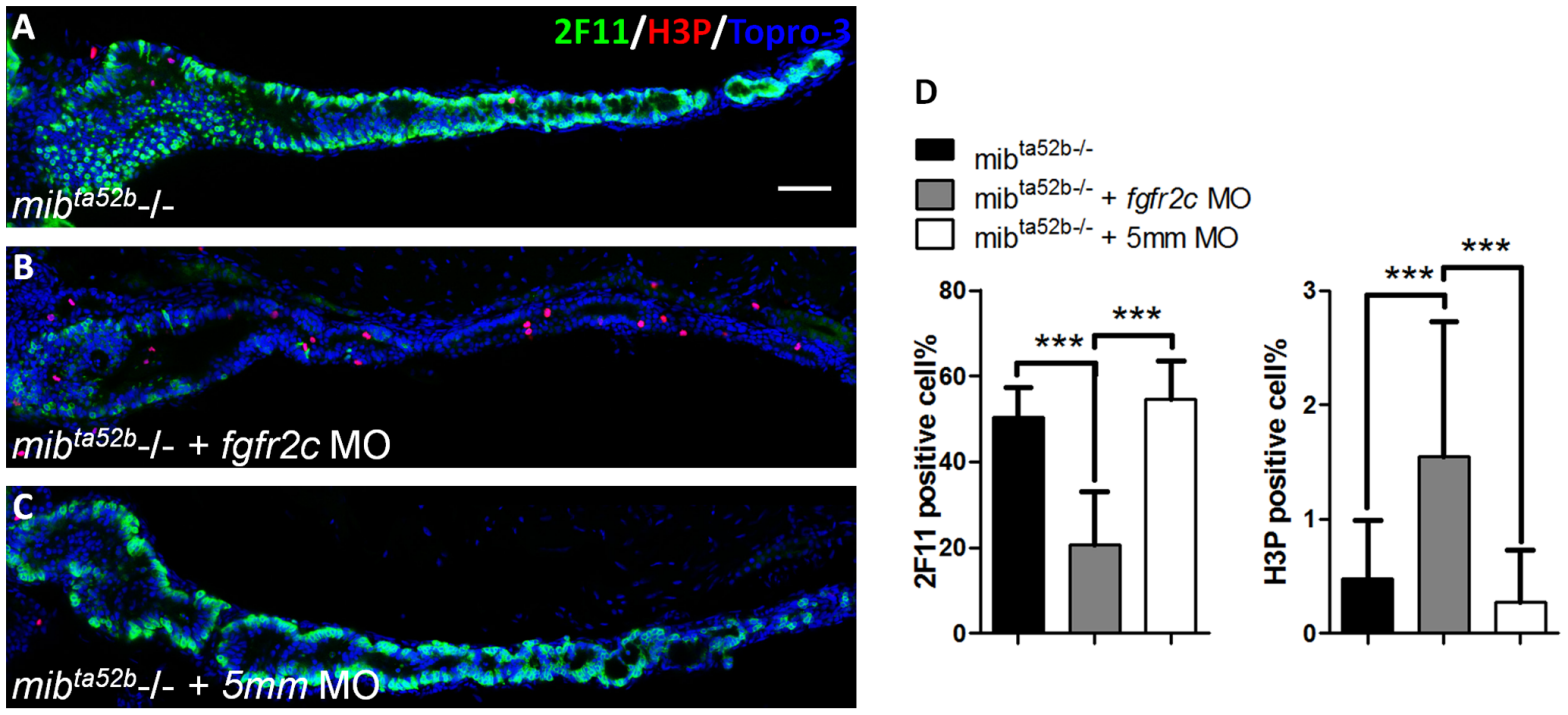
The secretory cell differentiation of *mib^ta52b^* mutants after injection with *fgfr2c* morpholino. The 2F11 (green) and H3P (red) antibodies were used to label the secretory and proliferating cells respectively, in (**A**) *mib^ta52b^* mutant, (**B**) mutant injected with *fgfr2c* morpholino, and (**C**) mutant injected with *fgfr2c-5* *mm* morpholino at 5 dpf. Topro-3 was used for nuclear counter staining (blue). (**D**) The bar charts show the percentages of secretory and proliferating cells. Error bars indicate SD. Scale bar = 50 µm.

It seemed likely that proliferating progenitor cells in *fgfr2c* morphants could not exit from cycling progression to undergo terminal differentiation. To test this hypothesis, we used the *mib^ta52b^* mutant to investigate the cell differentiation and proliferation. It was reported that Delta-Notch signaling is inhibited in *mib^ta52b^* mutant fish and differentiation of almost all intestinal cells is toward the secretory cell lineage [17], [47]. If Fgfr2c signaling were important for cell cycle exit and terminal cell differentiation, then 2F11 positive secretory cells should be reduced and M-phase cycling cells should be increased in *mib^ta52b^* mutants injected with *fgfr2c* morpholino. We observed a large ratio of 2F11 positive cells in *mib^ta52b^* and *mib^ta52b^* mutants injected with *fgfr2c*-5 mm morpholino at 5 dpf (50.5±6.8% and 54.6±9.3%, respectively; Fig. 8A and 8C). Less than one percent of H3P positive cycling cells could be detected in *mib^ta52b^* and *mib^ta52b^* mutants injected with *fgfr2c*-5 mm morpholino at 5 dpf (0.5±0.5% and 0.3±0.5%, respectively; Fig. 8A and 8C). There was a large reduction in secretory cells and an increased number of cycling cells in *mib^ta52b^* mutants injected with *fgfr2c* morpholino compared with control embryos at 5 dpf (2F11∶20.7±12.5%, *P*<0.001; H3P: 1.6±1.2%, *P*<0.001; Fig. 8B and 8D). We noticed that these H3P positive cells were not co-localized with the 2F11 signal. This indicated that proliferating cells were not differentiated secretory cells, but that they might be progenitor cells. Therefore, it is likely that intestinal proliferating progenitor cells cannot exit from cell cycling and are unable to differentiate further, in embryos that lack Fgfr2c signaling.

## Discussion

Intestinal progenitor cells are highly proliferative. It is important to understand what signaling factor(s) control these cycling cells’ differentiation to various mature intestinal epithelial cells, and how this is accomplished. In this study, we noted a novel role of Fgfr2c signaling in the gut epithelium. In the absence of Fgfr2c signaling, the differentiation of secretory and absorptive cells was inhibited, and cell proliferation was increased. Inhibition of Fgfr2c signaling in *mib^ta52b^* mutant embryos showed a similar effect. These results suggest that Fgfr2c signaling promotes progenitor cell’s exit from cell cycling, and their differentiation to various intestinal cell types.

In mice, *Fgfr1–3* is expressed in the developing gastrointestinal tract, including the forestomach, small intestine, colon, and cecum [22], [24], [27], [48], [49], [50]. Although few reports indicate that *Fgfr4* is expressed in gut tissue during developmental stages in mice, we found of *Fgfr4* in the stomach and intestine using the Genepaint database, which contains in situ hybridization data [51]. *Fgfr2b* ^−/−^ mice exhibit decreased differentiation gastric mucosa, and reduced numbers of proliferating epithelial cells [52]. *Fgfr3* ^−/−^ mice showed significantly reduced numbers of clonogenic stem cells and Paneth cells in the small intestine [27]. However, we found no reports that either Fgfr1 or Fgfr4 signaling regulates intestinal development and cell differentiation in mice.

We used zebrafish to study the function of Fgf signaling in the gut. Only *fgfr2* and *fgfr4* genes were strongly expressed in the intestine at 5 dpf, in which most of the observed cell differentiation occurred. In zebrafish, the *fgfr1* gene is duplicated to *fgfr1a* and *fgfr1b* [44]. RT-PCR did not reveal any evidence for the expression of *fgfr1b* at 5 dpf. Although *fgfr1a* was expressed in intestinal cells at 3 dpf, we did not detect its expression in intestinal mesenchymal or epithelial cells at 5 dpf. The *fgfr3* gene was not expressed in intestinal cells at 3 and 5 dpf. These expression patterns, and that for *fgfr3* in particular, were different from those reported for mice [27], [50].

In this study, we observed expression of *fgfr4* in the intestinal epithelial cells at both 5 dpf and adult fish (Fig. 2 and Fig. S2B). We administered *fgfr4* morpholino to verify whether or not Fgfr4 signaling affected cell differentiation. The results of *ifabp* WISH showed that loss of *fgfr4* expression does not disrupt intestinal cell differentiation (data not shown). Enterocyte expression of *Fgfr4* is important for bile acid homeostasis [53]. Thus, we suggest that Fgfr4 signaling might have the similar function in the zebrafish intestine.

The expressions of the two *Fgfr2* isoforms are highly regional specific. *Fgfr2b* and *Fgfr2c* are limited to epithelial and mesenchymal cells, respectively [54]. The *fgfr2* probe we used was full length over 4 kb cDNA for *fgfr2c* [36]. However, this probe was not specific to *fgfr2c* because there is only a 140 base difference between *fgfr2b* and *fgfr2c* in the full length region. We found that zebrafish *fgfr2* was expressed in both epithelial and mesenchymal cells of the intestine at 3 dpf, but that it was only expressed in intestinal mesenchymal cells at 5 dpf. This indicated that *fgfr2b* might only express in epithelial cells at 3 dpf, and that expression of *fgfr2c* is sustained in mesenchymal cells from 3 to 5 dpf. The *fgfr2* signal was restricted in mesenchymal cells of adult intestine tissue (Fig. S2A). We concluded that the major *fgfr2* isoform expressed in zebrafish intestine was *fgfr2c*, and that this gene specifically expressed in mesenchymal cells. However, *Fgfr2b* is reported to be the major isoform expressed in the developing gastrointestinal tract in mice [24], [48], [49]. Thus, Fgfr2 signaling may regulate intestinal cell proliferation and cell differentiation in different ways. In mice, Fgfr2b signaling acted autonomously in intestinal epithelium cells. Because *fgfr2c* was expressed in the mesenchymal cells of zebrafish intestine, other paracrine signal(s), regulated directly or indirectly by Fgfr2c may exist to control epithelial differentiation.

Mesenchymal-epithelial interactions are important for intestinal cell proliferation and differentiation. Many signaling factors are associated with these interactions, including Hedgehog (Hh) and Bmp factors [1]. The expression of *Ihh* and *Shh* could be detected in small intestinal epithelial cells and their receptors were observed in intestinal mesenchymal cells of mice; when Hh signaling was disrupted, the small intestinal epithelial cells were hyperproliferative, and these proliferating cells were negative for enterocyte differentiation [55]. Furthermore, with the exception of the proliferating zone, *Bmp4* was expressed in the mesenchymal layer of the small intestine, and *Bmpr1a* was expressed in the epithelial cells. In *Bmpr1a* conditional knockout mice, proliferating cells were generally increased in number [14]. In zebrafish, mesenchymal-epithelial interactions also regulate gut epithelial development. *MiR*-*145* is expressed in gut mesenchymal cells in zebrafish, while mesenchymal expressed *miR*-*145* directly controls *gata6* expression in the gut [56]. Recently reports suggest that *miR*-*145* regulates paracrine signaling to control gut development [57]. We found that *fgfr2c* is expressed in the mesenchymal layer, thus, some Fgf ligands must exist in the epithelial or mesenchymal layers of the zebrafish gut. Using RT-PCR, we identified putative Fgfr2c ligands (*fgf1*, *fgf2*, *fgf4*, *fgf17* and *fgf24*) that were expressed in 5 dpf zebrafish gut (data not shown), but we still do not know which specific Fgf ligands interact with Fgfr2c.

Wnt signaling is critical for intestine development. When Wnt ligands are present, cytosolic β-catenin proteins are not degraded by the destruction complex. Stable β-catenin proteins then translocate into the nucleus and bind with T cell factor (Tcf) transcription factors. This newly formed complex has transcriptional activity and can drive Wnt signaling target genes, including *c-Myc* and *cyclin D*, to promote cell cycle progression [6], [9]. In the *fgfr2c* morphant intestine, there were more proliferating cells present than there were in control embryos. We used β-catenin antibody to verify whether Wnt signaling is activated in the intestinal epithelial cells of *fgfr2c* morphants; only membrane accumulated β-catenin was detected, and there were no differences in the β-catenin localization pattern between *fgfr2c* morphants and the control embryos (data not shown). Thus, we still do not know whether Wnt signaling is activated in intestinal epithelial cells of *fgfr2c* morphants.

In conclusion, we found that Fgfr2c signaling derived from mesenchymal cells is important for regulating the differentiation of zebrafish intestine epithelial cells by promoting cell cycle exit. The results of Fgfr2c knockdown in *mib^ta52b^* mutants indicated that Fgfr2c signaling is required for intestinal cell differentiation. However, the mechanism by which mesenchymal cells control the behaviors of epithelial cell needs investigation. Furthermore, although the expression of *fgfr2* could be detected in the mesenchymal cells of adult zebrafish intestines, we do not know whether Fgfr2c signaling directs differentiation in adult fish.

## Supporting Information

Figure S1 The expression of *fgfrs* and the secretory cell differentiation of *fgfr2b* morphants. **(A)** *fgfr1a*, *fgfr2*, *fgfr3* and *fgfr4* gene were analyzed in 5 dpf zebrafish gut tissue by RT-PCR. **(C)** WT embryos and **(D)** *fgfr2b* morphants were double labeled using 2F11 antibody and WGA. The magnified image shows **(C’–D’)** enteroendocrine cells and **(C”–D”)** goblet cells. DAPI was used for nuclear counter staining (blue). **(B)** The bar charts show the percentages of 2F11 and WGA positive cells. Error bars indicate SD. Scale bar = 50 µm.
(TIF)

Figure S2 The expression of *fgfr2* and *fgfr4* in adult zebrafish intestine. Section in situ hybridization was used to analyze the expression of *fgfr2* and *fgfr4* genes. **(A)** *fgfr2* was detected in the lamina propria, and **(B)** *fgfr4* was expressed mainly in the epithelial layer of the intestine. Scale bar = 50 µm.
(TIF)

Table S1 Primer list for RT-PCR analysis.
(DOC)
