# Supplementary figures and images for: Fibroblast Growth Factor Receptor 2c Signaling Is Required for Intestinal Cell Differentiation in Zebrafish
Source: PLoS One. 2013 Mar 6;8(3):e58310. doi: 10.1371/journal.pone.0058310 (PMC3590179; doi:10.1371/journal.pone.0058310)

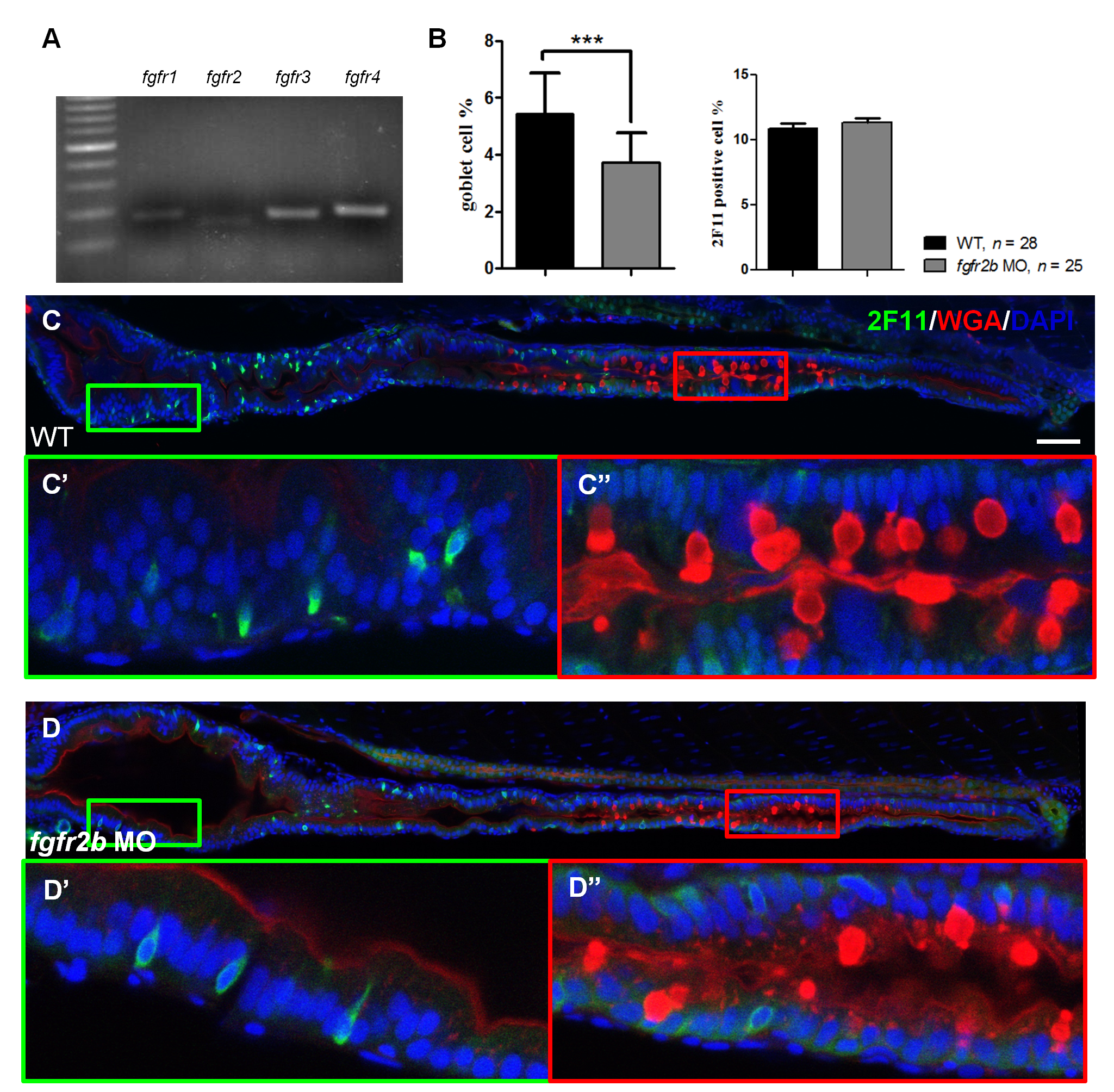

Supplement: Figure S1 — The expression of fgfrs and the secretory cell differentiation of fgfr2b morphants. (A) fgfr1a, fgfr2, fgfr3 and fgfr4 gene were analyzed in 5 dpf zebrafish gut tissue by RT-PCR. (C) WT embryos and (D) fgfr2b morphants were double labeled using 2F11 antibody and WGA. The magnified image shows (C’–D’) enteroendocrine cells and (C”–D”) goblet cells. DAPI was used for nuclear counter staining (blue). (B) The bar charts show the percentages of 2F11 and WGA positive cells. Error bars indicate SD. Scale bar = 50 µm. (TIF) [file pone.0058310.s001.tif]

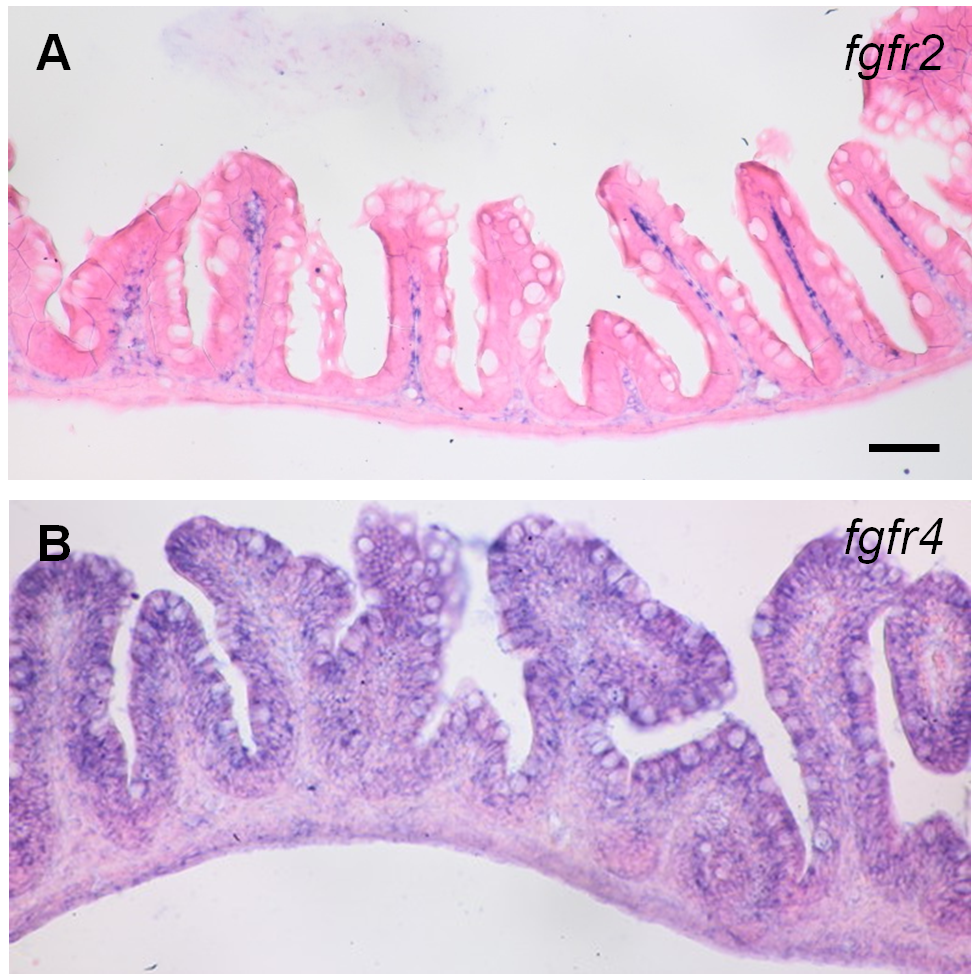

Supplement: Figure S2 — The expression of fgfr2 and fgfr4 in adult zebrafish intestine. Section in situ hybridization was used to analyze the expression of fgfr2 and fgfr4 genes. (A) fgfr2 was detected in the lamina propria, and (B) fgfr4 was expressed mainly in the epithelial layer of the intestine. Scale bar = 50 µm. (TIF) [file pone.0058310.s002.tif]
