# Supplementary material for: Fibroblast Growth Factor Receptor 2c Signaling Is Required for Intestinal Cell Differentiation in Zebrafish
Source: PLoS One. 2013 Mar 6;8(3):e58310. doi: 10.1371/journal.pone.0058310 (PMC3590179; doi:10.1371/journal.pone.0058310)
Supplement: Table S1 — Primer list for RT-PCR analysis. (DOC) [file pone.0058310.s003.doc]

Supplementary Table S1.

| Gene | Primer sequence (5’ to 3’) |
| --- | --- |
| *β-actine* | Forward: GTCCCTGTACGCCTCTGGTCG  Reverse: GCCGGACTCATCGTACTCCTG |
| *fgfr1a* | Forward: CTTGCTTTGCTCAGGGACTC  Reverse: CCGCATGTAGCTTCTTCTCC |
| *fgfr1b* | Forward: TGGGACTTCGCCAGATTATT  Reverse: GGCATGAAGCTTTTTCTCCA |
| *fgfr2* | Forward: GCACAAGCTCACCAAACAGA  Reverse: TCAGGGAGGTCGTATTCTGG |
| *fgfr3* | Forward: ATCGTATGGCAAAAAGACGG  Reverse: CAGCGAATCGATGACTTTGA |
| *fgfr4* | Forward: AGGGTGCTGGTGTCAATTTC  Reverse: TTAGGTCCATCCGAGAATGC |
| *ifabp* | Forward: AAGTCGACCGCAATGAGAAC  Reverse: GTTTGACATTGGGAGTGCAG |
